# miR-381-3p Cooperated With Hes1 to Regulate the Proliferation and Differentiation of Retinal Progenitor Cells

**DOI:** 10.3389/fcell.2022.853215

**Published:** 2022-02-25

**Authors:** Jiajing Wang, Na Sun, Yahan Ju, Ni Ni, Zhimin Tang, Dandan Zhang, Xiaochan Dai, Moxin Chen, Yiqi Wang, Ping Gu, Jing Ji

**Affiliations:** ^1^ Department of Ophthalmology, Ninth People’s Hospital, Shanghai Jiao Tong University School of Medicine, Shanghai, China; ^2^ Shanghai Key Laboratory of Orbital Diseases and Ocular Oncology, Shanghai, China; ^3^ Department of Ophthalmology, Xinhua Hospital, Shanghai Jiao Tong University School of Medicine, Shanghai, China; ^4^ Department of Ophthalmology, Zhongshan Hospital, Fudan University, Shanghai, China

**Keywords:** retinal progenitor cells, MiR-381-3p, Hes1, differentiation, proliferation

## Abstract

Retinal progenitor cells (RPCs) transplantation has become a promising therapy for retinal degeneration, which is a major kind of ocular diseases causing blindness. Since RPCs have limited proliferation and differentiation abilities toward retinal neurons, it is urgent to resolve these problems. MicroRNAs have been reported to have vital effects on stem cell fate. In our study, the data showed that overexpression of miR-381-3p repressed Hes1 expression, which promoted RPCs differentiation, especially toward neuronal cells, and inhibited RPCs proliferation. Knockdown of endogenous miR-381-3p increased Hes1 expression to inhibit RPCs differentiation and promote proliferation. In addition, a luciferase assay demonstrated that miR-381-3p directly targeted the Hes1 3’ untranslated region (UTR). Taken together, our study demonstrated that miR-381-3p regulated RPCs proliferation and differentiation by targeting Hes1, which provides an experimental basis of RPCs transplantation therapy for retinal degeneration.

## Introduction

Retinal degeneration is a primary kind of blindness-causing ocular disease worldwide, including age-related macular degeneration and retinitis pigmentosa, which can cause irreversible blindness in patients (Bourne et al., 2013). The pathogenesis of retinal degeneration is related to permanent damage and death of the photoreceptor cell layer (Lim et al., 2012). Retinal progenitor cells (RPCs), which have been discovered in adult mammalian eyes and even successfully extracted from the human retina, have the potential for self-renewal and differentiation toward retinal neuronal and glial lineages (Tropepe et al., 2000; Coles et al., 2004; Xia et al., 2012). However, the limited ability of RPCs to proliferate and differentiate toward retinal neurons hinders their future clinical application (Gu et al., 2007). Hence, finding a method to ameliorate the existing problems of RPCs proliferation and differentiation has become a matter of urgency.

MicroRNAs play essential roles in many biological processes, such as metabolism, development, proliferation, apoptosis, and stem cell differentiation (Krol et al., 2010). Based on current studies, a few microRNAs have been identified that can regulate RPCs proliferation and differentiation. For instance, miR-17 and miR-29a can reduce RPCs proliferation and promote differentiation (Zhang et al., 2017; Sun et al., 2020). Moreover, miR-762 can promote RPCs proliferation and inhibit differentiation (Gao et al., 2020). However, the role microRNAs play in RPCs fate is not thoroughly understood.

In our study, we demonstrated that miR-381-3p, the expression of which obviously increased with the process of RPCs differentiation, could regulate not only RPCs differentiation but also proliferation. TargetScan (http://www.targetscan.org) and miRDB (http://mirdb.org) were used to predict that Hes1 was a potential target of miR-381-3p. We sought to demonstrate that Hes1 is the direct target gene of miR-381-3p by a luciferase assay. Furthermore, we investigated the effect of Hes1 on RPCs fate. In general, our data explained that miR-381-3p inhibited RPCs proliferation and promoted RPCs differentiation, especially toward neuronal cells, by directly targeting Hes1. This study provided a new perspective and a better understanding of microRNA determination in RPCs fate.

## Materials and Methods

### Isolation, Culture and Differentiation of Retinal Progenitor Cells

RPCs were isolated from fresh retinal tissue of GFP transgenic postnatal Day 1 C57BL/6 mice. The assays were performed as previously described (Tang et al., 2019). In brief, retinal tissue was extracted under a type microscope after the eyeball was removed. Then we digested the tissue with TrypLE Express enzyme (Gibco) diluted five times and then centrifuged it. The cells were seeded in T25 flasks and cultured with proliferation medium containing advanced Dulbecco’s modified Eagle’s medium (DMEM)/F12 (Invitrogen, Carlsbad, CA, United States), 20 ng/ml recombinant epidermal growth factor (EGF, Invitrogen), 1% N2 neural supplement (Invitrogen), 2 mM L-glutamine (Invitrogen) and 100 U/mL penicillin-streptomycin. In differentiation studies, cells were cultured in differentiation medium containing 10% fetal bovine serum (FBS) (Invitrogen) without EGF. RPCs from third passage to fifth passage were used in this study. RPCs were cultured for 3 days in proliferation medium and for 7 days in differentiation medium.

All animals were treated according to the ARVO animal usage standards, and experimental protocols were approved by the Ethics Committee of the Ninth People’s Hospital affiliated with Shanghai Jiao Tong University School of Medicine.

### miRNA, siRNA Construction

miR-381-3p oligonucleotides (miR-381-3p mimics, miR-381-3p inhibitors and negative control) and small interfering RNAs (si-Hes1) were synthesized chemically by Zuorun Biotech Co., Ltd. (Shanghai, China). The oligonucleotides sequences of miR-381-3p mimics were: 5′-UAU​ACA​AGG​GCA​AGC​UCU​CUG​U-3′, of miR-381-3p inhibitors were: 5′-ACA​GAG​AGC​UUG​CCC​UUG​UAU​A-3′, of miR-381-3p negative control were: 5′-UUU​GUA​CUA​CAC​AAA​AGU​ACU​G-3’. The oligonucleotide sequences of si-Hes1 were: 5′-CCG​GCA​UUC​CAA​GCU​AGA​GAA​TT-3′.

### Transfection

RPCs were cultured with differentiation medium in 6-well plates for 12 h before transient transfection. A final concentration of 50 nM in Opti-MEM (Invitrogen) containing miR-381-3p mimics, miR-381-3p inhibitors, negative control and si-Hes1 was mixed with Lipofectamine 2000 (Invitrogen) in serum-free medium and incubated at room temperature for 20 min. Then, the cells were transfected for 6 h at 70% confluence. After transfection, the medium was renewed with fresh proliferation or differentiation medium. RPCs were repeatedly transfected every 3 days in differentiation culture conditions.

### Isolation, Quantification of Total RNA and Test of RNA Integrity

Total RNA was isolated from the cultured cells using Trizol reagent (TaKaRa, Japan) according to previously reported study (Tang et al., 2021a). The concentration and purity of total RNA were tested by spectrophotometry at optical density (OD) 260 and 280 nm. RNA samples whose OD260/280 ratio was between 1.9 and 2.1 were selected for cDNA synthesis. Moreover, we tested the integrity of the RNA by Eukaryote total RNA Nano assay on Agilent Bioanalyzer.

### Reverse Transcription and Quantitative Polymerase Chain Reaction

One thousand nanograms of total RNA was transcribed reversely into cDNA using the PrimeScript RT reagent kit protocol (Perfect Real Time; TaKaRa), and finally, a 20 μL reaction volume was synthesized (Tang et al., 2021b). cDNA was obtained using a miRcute miRNA first-strand cDNA synthesis kit (Tiangen Biotech Co., Ltd.). qPCR was performed to detect the expression of mRNA in a 10 μL final reaction volume including Power SYBR Green PCR Master Mix (Applied Biosystems). MicroRNA qPCR was conducted with 2*miRcute miRNA premix (Tiangen Biotech Co.). The relative mRNA or miRNA expression levels are expressed as the fold change relative to the controls after normalizing the expression of the reference gene (β-actin or U6). These primer sequences are listed in Supplementary Table S1.

### Western Blot Analysis

The protein was extracted after 3 days under proliferation culture, and extracted after 7 days under differentiation culture. RIPA (pH 7.4, 50 mM Tris–HCl, 150 mM NaCl, 1 mM EDTA, 1 mM Na_3_VO_4_, 1% Triton X-100, 0.1% SDS, 5 mM PMSF) was used to extract total proteins on ice. Then the concentration of total proteins was detected by BCA protein kit (Thermo Scientific, United States). Proteins were separated by sodium dodecyl sulfate–polyacrylamide gel electrophoresis (SDS–PAGE) and transferred to PVDF membranes (Millipore, Billerica, MA, United States). The membranes were blocked with 5%BSA for 1 h, and then incubated with the following primary antibodies, respectively: mouse anti-β-actin (Proteintech; 1:5,000), rabbit monoclonal anti-Hes1 (ABclonal; 1:1,000), rabbit polyclonal anti-β3-tubulin (Abcam; 1:1,000), rabbit polyclonal anti-PKC-α (Proteintech; 1:1,000) at 4°C for 8 h. After adding the respective secondary antibodies to the membranes, protein expression was visualized by an ECL Plus Western Blot Detection Kit (Tanton).

### EdU Assays

EdU assay was used to assess cell proliferation using an *in vitro* EdU Kit (RiboBio, China), following the manufacturer protocol. In brief, the assay was performed in 24-well format, with EdU reagent being added to proliferating cells transfected with miR-381-3p mimics, inhibitors, or control for 8 h. Then RPCs were fixed with 4% paraformaldehyde (PFA) for 30 min at room temperature and stained with Apollo Dye Solution. Then, nuclei acids were incubated with Hoechst 33,342 for 30 min. The cells were observed by fluorescence microscopy (Olympus BX51, Japan).

### Immunocytochemistry Analysis

Immunocytochemistry analysis was conducted to evaluate the differentiation ability of RPCs that were plated on glass coverslips (VWR, West Chester, PA, United States) in 24-well plates with differentiation medium. At the right time after RPCs were transfected with miR-381-3p mimics, miR-381-3p inhibitors, or si-Hes1, RPCs were fixed by using 4% PFA (Sigma–Aldrich) for 1 h at room temperature. Then, RPCs were blocked with PBS containing 10% goat serum (Gibco) and permeabilized with 0.3% Triton X-100 (Sigma–Aldrich) for 1 h. Subsequently, RPCs were incubated for 8 h at 4°C with rabbit polyclonal anti-β3-tubulin (Abcam; 1:200) and rabbit polyclonal anti-PKC-α (Proteintech; 1:200) antibodies. Afterward, the cells were incubated for 1 h at room temperature with fluorescently labeled anti-rabbit antibodies (BD Biosciences, 1:800). Hoechst (Invitrogen) was used to stain the cell nucleus. The images of immunoreactive RPCs were filmed by fluorescence microscopy (Olympus BX51, Japan).

### Luciferase Assay

The procedures of luciferase assay were conducted as described above (Wang et al., 2018). In brief, the Hes1 3′UTR fragments, including putative miR-381-3p binding sites (positions 183–190), were inserted into luciferase reporter sequences. Then, luciferase reporter vectors were cotransfected into HEK293 cells with miR-381-3p mimics or miR-NC. After 48 h, the luciferase activity of cells was detected by a Dual Luciferase Reporter Assay System (Zuorun Biotech Co., Ltd., China) according to the manufacturer’s protocol.

### Statistical Analysis

All data are shown as the mean ± standard deviation (SD). Each experiment was performed at least three times, unless otherwise specified. Statistical significance was analyzed by Student’s t-tests or one-way ANOVA using GraphPad Prism 7.0 software. Differences were considered statistically significant when *p* < 0.05.

## Results

### Endogenous Expression Levels of miR-381-3p and Hes1 in the Process of Retinal Progenitor Cells Differentiation

**FIGURE 1:**
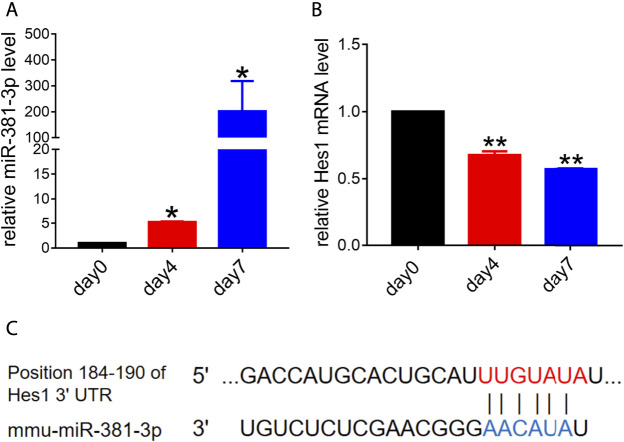
Endogenous expression levels of miR-381-3p and Hes1 in the process of RPCs differentiation. **(A,B)** qPCR results showed that the expression of miR-381-3p gradually increased while the expression of Hes1 gradually decreased during RPCs differentiation. **(C)** TargetScan and miRDB predicted the 3′UTR of Hes1 as a potential target of miR-381-3p. Day 0, which represented the undifferentiated RPCs state, was used as a normalizer. Error bars represent the mean standard deviation for *n* = 3 independent experiments. *p < 0.05, **p < 0.01 (one-way ANOVA).

Previous studies suggested that miR-381 had a great effect on cell growth, migration or apoptosis (Jin et al., 2020; Yang et al., 2020; Fang et al., 2021). To determine the role that miR-381 plays in RPCs, we first evaluated the endogenous expression levels of miR-381-3p during RPCs differentiation. qPCR analysis showed that miR-381-3p expression increased dramatically with the process of RPCs differentiation, which finally reached approximately 200-fold at the seventh day compared to the expression at the beginning (Figure 1A). In contrast, the expression of Hes1, which is a potential target gene of miR-381-3p according to the prediction of both TargetScan and miRDB, decreased gradually with the RPCs differentiation process. The expression of Hes1 mRNA at day 7 was reduced by approximately 0.5-fold compared to that at day 0 (Figures 1B,C). These data implied that miR-381-3p and Hes1 had an underlying negative correlation during RPCs differentiation and that both of them could regulate RPCs fate.

### miR-381-3p Inhibits Retinal Progenitor Cells Proliferation and Promotes Retinal Progenitor Cells Differentiation

**FIGURE 2:**
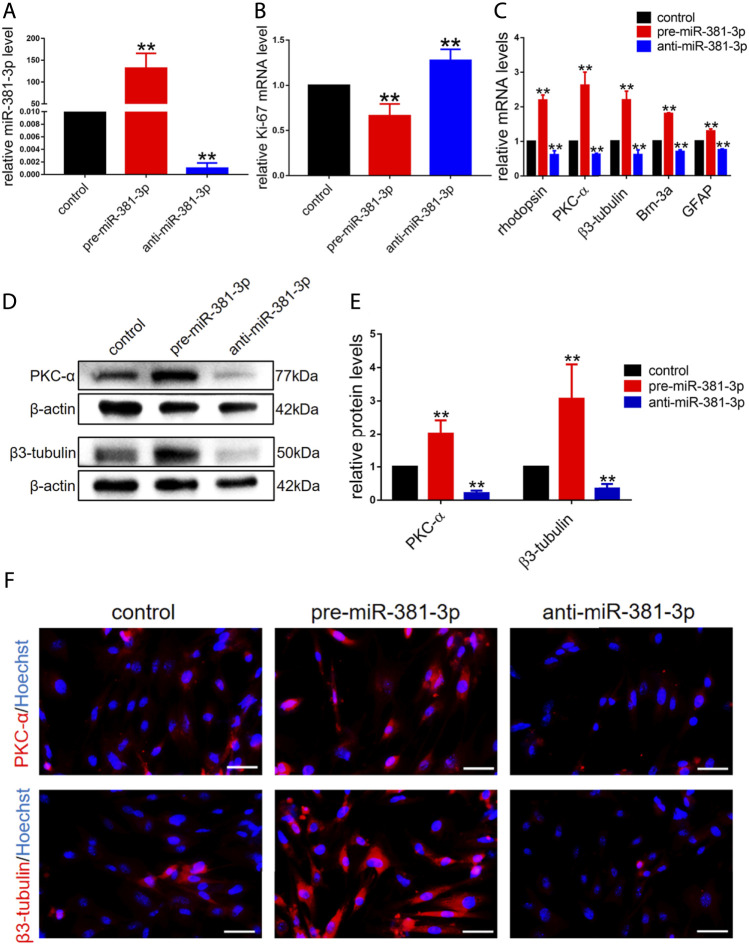
miR-381-3p inhibits RPCs proliferation and promotes RPCs differentiation. **(A)** qPCR results showed that the expression of miR-381-3p was markedly increased by transfection with mimics and decreased by inhibitors treatment. **(B)** qPCR results showed that the expression of Ki-67 was reduced in RPCs transfected with mimics and increased in RPCs transfected with inhibitors. **(C)** qPCR showed that the expression levels of differentiation-related markers, including rhodopsin, PKC-α, β3-tubulin, Brn-3a and GFAP, were upregulated by mimics and downregulated by inhibitors. **(D,E)** Western blot results showed that the expression levels of PKC-α and β3-tubulin were increased by transfection with mimics and reduced by transfection with inhibitors. **(F)** Immunocytochemistry assessment results were consistent with the western blot results. Western blot bands were normalized to β-actin. Scale bars: 50 µm. Error bars represent the mean standard deviation for *n* = 3 independent experiments. *p < 0.05, **p < 0.01 (one-way ANOVA).

To investigate whether miR-381-3p had an effect on RPCs proliferation and differentiation, RPCs were transfected with miR-381-3p mimics or inhibitors and then cultured in proliferation or differentiation medium. miR-381-3p expression in the pre-miR-381-3p group was increased nearly 150-fold, while that in the anti-miR-381-3p group was decreased nearly 0.002-fold compared to that in the controls, thus showing great transfection efficiency (Figure 2A). We next evaluated the proliferation ability of RPCs by qPCR. As shown in Figure 2B, Ki-67 (proliferative marker) expression was reduced in the miR-381-3p mimic group but increased in the inhibitor group. EdU assays also showed that miR-381-3p mimics attenuated proliferation in cells while miR-381-3p inhibitors significantly promoted proliferation (Supplementary Figure S1).

To investigate the effect of miR-381-3p on RPCs differentiation, qPCR, western blot and immunocytochemistry experiments were carried out. The gene expression of retinal neuronal cell markers, including rhodopsin (photoreceptor marker), PKC-α (bipolar neuron marker), β3-tubulin (panneuronal marker) and Brn-3a (marker of ganglion cells), increased by approximately 2-fold, while the expression of glial cell marker (GFAP) increased by approximately 1.3-fold in pre-miR-381-3p treated RPCs compared to the controls. Moreover, the expression of these markers in anti-miR-381-3p-treated RPCs was downregulated (Figure 2C). In addition, western blot analysis revealed that pre-miR-381-3p could significantly increase the protein levels of PKC-α and β3-tubulin. In contrast, the miR-381-3p inhibitors had the opposite effect on these neuronal cell markers (Figures 2D,E). The immunocytochemistry results were consistent with the western blot results (Figure 2F). Taken together, these results suggested that miR-381-3p obviously promotes RPCs differentiation toward retinal nerve cells, especially neuronal cells.

### Knockdown of Endogenous Hes1 Promotes Retinal Progenitor Cells Differentiation and Inhibits Retinal Progenitor Cells Proliferation

**FIGURE 3:**
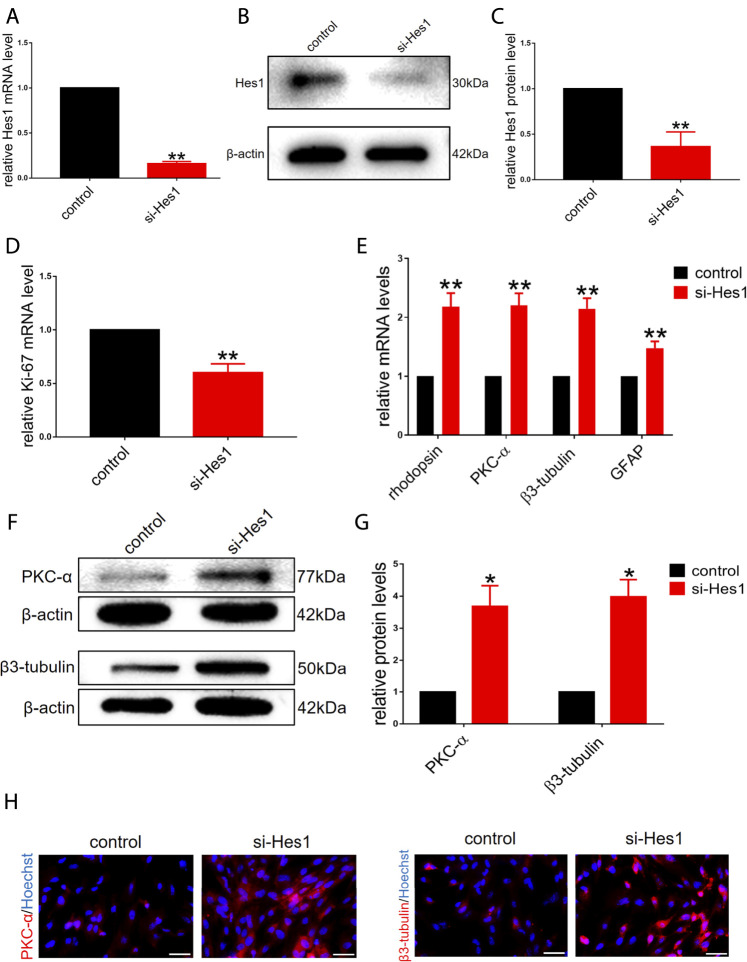
Knockdown of endogenous Hes1 promotes RPCs differentiation and inhibits RPCs proliferation. **(A–C)** qPCR and western blot results showed that the expression of Hes1 was significantly decreased by transfection with si-Hes1. **(D)** qPCR results showed that the expression of Ki-67 was inhibited by transfection with si-Hes1. **(E)** According to the qPCR results, the expression levels of differentiation-related markers, including rhodopsin, PKC-α, β3-tubulin and GFAP, were markedly upregulated in the si-Hes1-treated group. **(F,G)** Western blot results showed that the expression of PKC-α and β3-tubulin was increased in the si-Hes1 groups compared to the controls. (H) Immunocytochemistry assessment results were consistent with the western blot results. Western blot bands were normalized to β-actin. Scale bars: 50 µm. Error bars represent the mean standard deviation for *n* = 3 independent experiments. *p < 0.05, **p < 0.01 (one-way ANOVA or Student’s t-tests).

Based on the gradual reduction in the expression of endogenous Hes1 in RPCs during the onset of differentiation (Figure 1B), we next sought to determine whether Hes1 plays a potential role in RPCs fate. We transfected si-Hes1 into RPCs to knock down endogenous Hes1 and examined the knockdown efficiency. The qPCR and western blot analyses revealed a dramatic reduction in the mRNA and protein expression levels of Hes1 in RPCs treated with si-Hes1 compared to controls (Figures 3A–C). After culturing RPCs in proliferation medium for 3 days, the gene expression of Ki-67 was markedly decreased in the si-Hes1 groups (Figure 3D), which indicated that repression of Hes1 inhibited RPCs proliferation.

Next, we investigated the effect of Hes1 on RPCs differentiation. In RPCs treated with si-Hes1, there was a significant increase in the expression of differentiation-related markers, including rhodopsin, PKC-α, β3-tubulin and GFAP, and the increase in the expression of retinal neuronal markers was markedly higher than that in glial cell markers (Figure 3E). This result illustrated that si-Hes1 can enhance RPCs differentiation and make RPCs tend to differentiate to retinal neuronal cells. To confirm this result, we detected the expression of PKC-α and β3-tubulin by western blot and immunostaining analyses. The results were consistent with the qPCR results (Figures 3F–H). Above all, these results indicated that Hes1 negatively regulated RPCs differentiation and positively regulated proliferation.

### Hes1 Is a Functional Target Gene of miR-381-3p in Retinal Progenitor Cells

**FIGURE 4:**
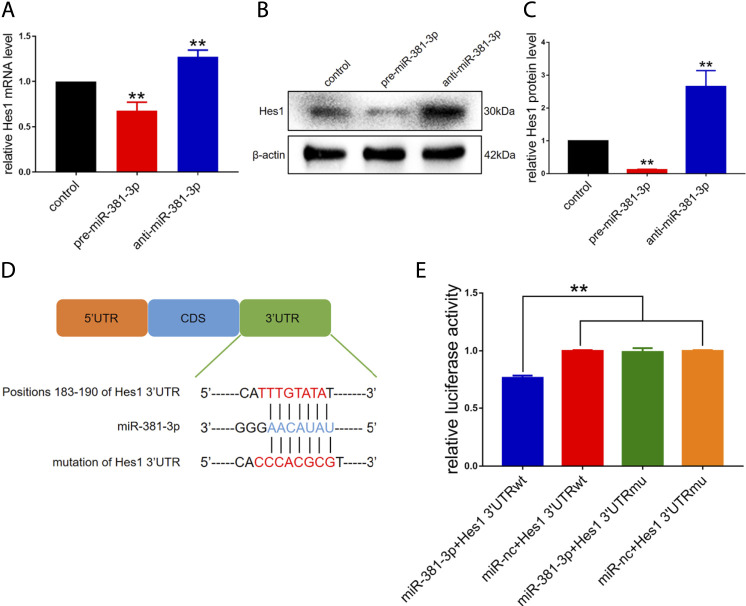
Hes1 is a functional target gene of miR-381-3p in RPCs. **(A–C)** qPCR and western blot results showed that Hes1 expression was downregulated by miR-381-3p mimics and upregulated by inhibitors. **(D)** Positions 183–190 of the 3′UTR of Hes1 mRNA or mutated 3′UTR sequences were designed and inserted into the pGL3 control plasmids. **(E)** Luciferase assay showed that the relative luciferase activity was diminished by cotransfection of miR-381-3p and Hes1 3′UTR-wt compared to the other groups, while cotransfection of miR-381-3p and Hes1 3′UTR-mu had nearly no effect on luciferase activity. Western blot bands were normalized to β-actin. Error bars represent the mean standard deviation for *n* = 3 independent experiments. *p < 0.05, **p < 0.01 (one-way ANOVA).

As shown in Figure 1, miR-381-3p and Hes1 were negatively correlated during RPCs differentiation. Moreover, both TargetScan and miRDB predicted that miR-381-3p would interact with Hes1. According to these findings, we hypothesized that miR-381-3p targets Hes1 to affect RPCs fate. To verify this hypothesis, we first assessed the mRNA and protein levels of Hes1 in the pre-miR-381-3p and anti-miR-381-3p groups. The expression of Hes1 mRNA was downregulated in RPCs with miR-381-3p mimics and upregulated in RPCs with inhibitors (Figure 4A). Meanwhile, the protein levels of Hes1 were markedly decreased in the pre-miR-381-3p groups and increased in the anti-miR-381-3p groups compared to the controls (Figures 4B,C). This phenomenon suggested that miR-381-3p repressed the protein expression of Hes1 by interacting with its mRNA.

To confirm that miR-381-3p directly binds to the Hes1 mRNA 3′UTR to posttranscriptionally repress Hes1 expression, a luciferase assay was performed. Luciferase reporter vectors carrying the sequences of the Hes1 3′UTR at positions 183–190, including either the putative miR-381-3p binding site (called Hes1 3′UTR-wt) or its mutant sequences (called Hes1 3′UTR-mu), were constructed to verify the direct interaction (Figure 4D). The relative luciferase activity was diminished by cotransfection of miR-381-3p and Hes1 3′UTR-wt compared to other groups, while cotransfection of miR-381-3p and Hes1 3′UTR-mu had nearly no effect on luciferase activity (Figure 4E). Taken together, miR-381-3p repressed the expression of Hes1 by directly targeting the Hes1 3′UTR.

In summary, these results demonstrated that miR-381-3p inhibited RPCs proliferation and promoted differentiation by targeting Hes1 through direct binding to its 3′UTR.

## Discussion

Currently, RPCs, embryonic stem cells (ESCs), induced pluripotent stem cells (iPSCs) and mesenchymal stem cells (MSCs) are research hotspots in retinal degeneration stem cell therapy. However, ESCs present low efficiency of transformation and ethical issues (Rivron et al., 2018); iPSC induced differentiation depends on the virus and has potential tumorigenicity (Gao et al., 2016); MSC transplantation has complications, etc. (Park et al., 2017). Comparatively, RPCs do not present these problems, which makes RPCs the preferred cell source for ophthalmic stem cell therapy (Li et al., 2009; Zhou et al., 2015). However, RPCs present a poor ability to proliferate and differentiate into retinal neurons, and these problems must be resolved. Efficient methods have been researched to address the abovementioned problems, such as supplementation with factors, including ciliary neurotrophic factors, and the use of microRNAs and retinal tissue engineering using hyaluronan (Tang et al., 2017). However, ciliary neurotrophic factor treatment leads to serious peripheral side effects (Pasquin et al., 2015), and retinal tissue engineering using artificial materials requires testing for biocompatibility, biotoxicity and mechanical properties. In comparison, microRNAs represent excellent mimics for the biological production of retinal cells and have no biotoxicity. Moreover, the self-renewing properties of stem cells are regulated by intracellular mechanisms, including differential gene expression controlled at epigenetic, translational and posttranslational levels, and microRNAs play important roles in regulating gene expression and stem cell fate (Gangaraju and Lin, 2009). Hence, the identification of microRNAs that can modulate the proliferation and differentiation of RPCs obviously has a great impact on stem cell therapy (Fan et al., 2016).

miR-381 belongs to the miR-154 gene family and is encoded on the 14q32.31 chromosomal region, where microRNAs have been discovered to regulate tumorigenicity (Rothschild et al., 2012). Indeed, miR-381 has been reported to suppress tumors by repressing cancer cell proliferation in various cancer types, such as breast cancer (Xue et al., 2017), pancreatic cancer (Qiao et al., 2019), and cervical cancer (Liu et al., 2018). Furthermore, previous studies demonstrated that miR-381 also had an impact on biological function in noncancerous conditions. For example, miR-381 favors the repair of nerve injury (Xue et al., 2020). However, few studies on miR-381 have focused on its impact in noncancerous conditions. Since retinal tissue belongs to nervous tissue and the effect of miR-381 on RPCs fate has not been determined, we explored whether miR-381 regulates the proliferation and differentiation of RPCs. In this study, we found that miR-381-3p, the expression of which increased obviously with the process of RPCs differentiation, had a great impact on RPCs proliferation and differentiation. Our data illustrated that miR-381-3p especially increased the expression of these differentiation-related markers including rhodopsin, PKC-α, β3-tubulin and Brn-3a, and decreased the expression of Ki-67, which indicated that miR-381-3p promoted RPCs differentiation, especially toward neuronal cells, and inhibited RPCs proliferation.

To determine the mechanism underlying the mediation of RPCs proliferation and differentiation by miR-381-3p, we predicted and validated the possible target genes of miR-381-3p. According to the predictions of TargetScan and miRDB, Hes1 was revealed to be a potential target of miR-381-3p. Additionally, the expression of miRNA target gene should be at high levels when miRNA expression is at a low level (Ni et al., 2014). Our data showed a remarkable negative correlation between miR-381-3p and Hes1 during RPCs differentiation. Together with the data that demonstrated that overexpression of miR-381-3p repressed the expression of Hes1 at the mRNA and protein levels, we hypothesized that Hes1 was the direct target gene of miR-381-3p. Since microRNA binding sites in animal mRNA occur at the 3′UTR (Carthew and Sontheimer, 2009), a luciferase assay was conducted, and the data proved that miR-381-3p modulates Hes1 expression by directly targeting its 3′UTR.

Hes1, the transcriptional repressor Hairy Enhancer of Split 1, is a member of the hairy-related basic helix-loop-helix (bHLH) family (Kageyama et al., 2007). Hes1 is an evolutionarily conserved target of Notch signaling which regulates neurogenesis timing and progenitor cell population dynamics in the course of mouse retinal development (Jarriault et al., 1995). A previous study found that all cells expressed Hes1 initially during the development of the eye. Hes1 maintains proliferation while blocking retinal ganglion cell and bipolar neuron formation throughout retinal neurogenesis, indicating that Hes1 plays an important role in retinal development (Bosze et al., 2020). Consistent with previous studies, we found that knockdown of Hes1 significantly regulated RPCs fate by promoting RPCs differentiation to retinal neuronal cells and inhibiting proliferation.

In summary, our study illustrated that miR-381-3p regulated RPCs proliferation and differentiation by directly targeting Hes1, thus providing a more comprehensive understanding of the molecular mechanism underlying RPCs fate and new inspiration for RPCs transplantation treatment for retinal degeneration. Further studies on the functions of miR-381-3p and Hes1 *in vivo* will attract much interest in this field.

## Data Availability

The original contributions presented in the study are included in the article/Supplementary Material, further inquiries can be directed to the corresponding authors.

## References

[B1] Bosze B. Moon M. S. Kageyama R. Brown N. L. (2020). Simultaneous Requirements for Hes1 in Retinal Neurogenesis and Optic Cup-Stalk Boundary Maintenance. J. Neurosci. 40 (7), 1501–1513. 10.1523/JNEUROSCI.2327-19.2020 31949107 PMC7044741

[B2] Bourne R. R. A. Stevens G. A. White R. A. Smith J. L. Flaxman S. R. Price H. (2013). Causes of Vision Loss Worldwide, 1990-2010: a Systematic Analysis. Lancet Glob. Health 1 (6), e339–e349. 10.1016/s2214-109x(13)70113-x 25104599

[B3] Carthew R. W. Sontheimer E. J. (2009). Origins and Mechanisms of miRNAs and siRNAs. Cell 136 (4), 642–655. 10.1016/j.cell.2009.01.035 19239886 PMC2675692

[B4] Coles B. L. K. Angenieux B. Inoue T. Del Rio-Tsonis K. Spence J. R. McInnes R. R. (2004). Facile Isolation and the Characterization of Human Retinal Stem Cells. Proc. Natl. Acad. Sci. 101 (44), 15772–15777. 10.1073/pnas.0401596101 15505221 PMC524825

[B5] Fan Y. Siklenka K. Arora S. K. Ribeiro P. Kimmins S. Xia J. (2016). miRNet - Dissecting miRNA-Target Interactions and Functional Associations through Network-Based Visual Analysis. Nucleic Acids Res. 44 (W1), W135–W141. 10.1093/nar/gkw288 27105848 PMC4987881

[B6] Fang H. Li H. F. Yan J. Y. Yang M. Zhang J. P. (2021). Dexmedetomidine‐up‐regulated microRNA‐381 Exerts Anti‐inflammatory Effects in Rats with Cerebral Ischaemic Injury via the Transcriptional Factor IRF4. J. Cel Mol. Med. 25 (4), 2098–2109. 10.1111/jcmm.16153 PMC7882963 33314611

[B7] Gangaraju V. K. Lin H. (2009). MicroRNAs: Key Regulators of Stem Cells. Nat. Rev. Mol. Cel Biol. 10 (2), 116–125. 10.1038/nrm2621 PMC4118578 19165214

[B8] Gao M. Yao H. Dong Q. Zhang H. Yang Z. Yang Y. (2016). Tumourigenicity and Immunogenicity of Induced Neural Stem Cell Grafts versus Induced Pluripotent Stem Cell Grafts in Syngeneic Mouse Brain. Sci. Rep. 6, 29955. 10.1038/srep29955 27417157 PMC4945932

[B9] Gao H. Ni N. Zhang D. Wang Y. Tang Z. Sun N. (2020). miR-762 Regulates the Proliferation and Differentiation of Retinal Progenitor Cells by Targeting NPDC1. Cell Cycle 19 (14), 1754–1767. 10.1080/15384101.2020.1777805 32544377 PMC7469545

[B10] Gu P. Harwood L. J. Zhang X. Wylie M. Curry W. J. Cogliati T. (2007). Isolation of Retinal Progenitor and Stem Cells from the Porcine Eye. Mol. Vis. 13, 1045–1057. 17653049 PMC2776542

[B11] Jarriault S. Brou C. Logeat F. Schroeter E. H. Kopan R. Israel A. (1995). Signalling Downstream of Activated Mammalian Notch. Nature 377 (6547), 355–358. 10.1038/377355a0 7566092

[B12] Jin D. Guo J. Wu Y. Chen W. Du J. Yang L. (2020). Metformin-repressed miR-381-YAP-Snail axis Activity Disrupts NSCLC Growth and Metastasis. J. Exp. Clin. Cancer Res. 39 (1), 6. 10.1186/s13046-019-1503-6 31906986 PMC6945774

[B13] Kageyama R. Ohtsuka T. Kobayashi T. (2007). The Hes Gene Family: Repressors and Oscillators that Orchestrate Embryogenesis. Development 134 (7), 1243–1251. 10.1242/dev.000786 17329370

[B14] Krol J. Loedige I. Filipowicz W. (2010). The Widespread Regulation of microRNA Biogenesis, Function and Decay. Nat. Rev. Genet. 11 (9), 597–610. 10.1038/nrg2843 20661255

[B15] Li S. Y. Yin Z. Q. Chen S. J. Chen L.-F. Liu Y. (2009). Rescue from Light-Induced Retinal Degeneration by Human Fetal Retinal Transplantation in Minipigs. Curr. Eye Res. 34 (7), 523–535. 10.1080/02713680902936148 19899965

[B16] Lim L. S. Mitchell P. Seddon J. M. Holz F. G. Wong T. Y. (2012). Age-related Macular Degeneration. Lancet 379 (9827), 1728–1738. 10.1016/s0140-6736(12)60282-7 22559899

[B17] Liu C. Tian X. Zhang J. Jiang L. (2018). Long Non-coding RNA DLEU1 Promotes Proliferation and Invasion by Interacting with miR-381 and Enhancing HOXA13 Expression in Cervical Cancer. Front. Genet. 9, 629. 10.3389/fgene.2018.00629 30581456 PMC6292861

[B18] Ni N. Zhang D. Xie Q. Chen J. Wang Z. Deng Y. (2014). Effects of Let-7b and TLX on the Proliferation and Differentiation of Retinal Progenitor Cells *In Vitro* . Sci. Rep. 4, 6671. 10.1038/srep06671 25327364 PMC4202307

[B19] Park S. S. Moisseiev E. Bauer G. Anderson J. D. Grant M. B. Zam A. (2017). Advances in Bone Marrow Stem Cell Therapy for Retinal Dysfunction. Prog. Retin. Eye Res. 56, 148–165. 10.1016/j.preteyeres.2016.10.002 27784628 PMC5237620

[B20] Pasquin S. Sharma M. Gauchat J.-F. (2015). Ciliary Neurotrophic Factor (CNTF): New Facets of an Old Molecule for Treating Neurodegenerative and Metabolic Syndrome Pathologies. Cytokine Growth Factor. Rev. 26 (5), 507–515. 10.1016/j.cytogfr.2015.07.007 26187860

[B21] Qiao G. Li J. Wang J. Wang Z. Bian W. (2019). miR-381 Functions as a Tumor Suppressor by Targeting ETS1 in Pancreatic Cancer. Int. J. Mol. Med. 44 (2), 593–607. 10.3892/ijmm.2019.4206 31173154 PMC6605709

[B22] Rivron N. Pera M. Rossant J. Martinez Arias A. Zernicka-Goetz M. Fu J. (2018). Debate Ethics of Embryo Models from Stem Cells. Nature 564 (7735), 183–185. 10.1038/d41586-018-07663-9 30542177

[B23] Rothschild S. I. Tschan M. P. Jaggi R. Fey M. F. Gugger M. Gautschi O. (2012). MicroRNA-381 Represses ID1 and Is Deregulated in Lung Adenocarcinoma. J. Thorac. Oncol. 7 (7), 1069–1077. 10.1097/jto.0b013e31824fe976 22592211

[B24] Sun N. Zhang D. Ni N. Tang Z. Gao H. Ju Y. (2020). miR-17 Regulates the Proliferation and Differentiation of Retinal Progenitor Cells by Targeting CHMP1A. Biochem. Biophys. Res. Commun. 523 (2), 493–499. 10.1016/j.bbrc.2019.11.108 31894018

[B25] Tang Z. Zhang Y. Wang Y. Zhang D. Shen B. Luo M. (2017). Progress of Stem/progenitor Cell-Based Therapy for Retinal Degeneration. J. Transl. Med. 15 (1), 99. 10.1186/s12967-017-1183-y 28486987 PMC5424366

[B26] Tang Z. Jiang F. Zhang Y. Zhang Y. Yuan Y. Huang X. (2019). Mussel-inspired Injectable Hydrogel and its Counterpart for Actuating Proliferation and Neuronal Differentiation of Retinal Progenitor Cells. Biomaterials 194, 57–72. 10.1016/j.biomaterials.2018.12.015 30583149

[B27] Tang Z. Ju Y. Dai X. Ni N. Liu Y. Zhang D. (2021). HO-1-mediated Ferroptosis as a Target for protection against Retinal Pigment Epithelium Degeneration. Redox Biol. 43, 101971. 10.1016/j.redox.2021.101971 33895485 PMC8099560

[B28] Tang Z. Huo M. Ju Y. Dai X. Ni N. Liu Y. (2021). Nanoprotection against Retinal Pigment Epithelium Degeneration via Ferroptosis Inhibition. Small Methods 5 (12), e2100848. 10.1002/smtd.202100848 34928015

[B29] Tropepe V. Coles B. L. K. Chiasson B. J. Horsford D. J. Elia A. J. McInnes R. R. (2000). Retinal Stem Cells in the Adult Mammalian Eye. Science 287 (5460), 2032–2036. 10.1126/science.287.5460.2032 10720333

[B30] Wang Y. Zhang D. Tang Z. Zhang Y. Gao H. Ni N. (2018). REST, Regulated by RA through miR-29a and the Proteasome Pathway, Plays a Crucial Role in RPC Proliferation and Differentiation. Cell Death Dis. 9 (5), 444. 10.1038/s41419-018-0473-5 29670089 PMC5906654

[B31] Xia J. Liu H. Fan X. Hu Y. Zhang Y. Wang Z. (2012). An *In Vitro* Comparison of Two Different Subpopulations of Retinal Progenitor Cells for Self-Renewal and Multipotentiality. Brain Res. 1433, 38–46. 10.1016/j.brainres.2011.11.054 22177772

[B32] Xue Y. Xu W. Zhao W. Wang W. Zhang D. Wu P. (2017). miR-381 Inhibited Breast Cancer Cells Proliferation, Epithelial-To-Mesenchymal Transition and Metastasis by Targeting CXCR4. Biomed. Pharmacother. 86, 426–433. 10.1016/j.biopha.2016.12.051 28012397

[B33] Xue Y. Xu T. Jiang W. (2020). Dexmedetomidine Protects PC12 Cells from Ropivacaine Injury through miR-381/LRRC4/SDF-1/CXCR4 Signaling Pathway. Regener. Ther. 14, 322–329. 10.1016/j.reth.2020.03.001 PMC7243045 32467829

[B34] Yang G. He F. Duan H. Shen J. Dong Q. (2020). lncRNA FLVCR-AS1 Promotes Osteosarcoma Growth by Targeting miR381-3p/CCND1. Onco Targets Ther. 13, 163–172. 10.2147/ott.s214813 32021264 PMC6966140

[B35] Zhang Y. Shen B. Zhang D. Wang Y. Tang Z. Ni N. (2017). miR-29a Regulates the Proliferation and Differentiation of Retinal Progenitors by Targeting Rbm8a. Oncotarget 8 (19), 31993–32008. 10.18632/oncotarget.16669 28404883 PMC5458264

[B36] Zhou P.-Y. Peng G.-H. Xu H. Yin Z. Q. (2015). c-Kit+ Cells Isolated from Human Fetal Retinas Represent a New Population of Retinal Progenitor Cells. J. Cel Sci. 128 (11), 2169–2178. 10.1242/jcs.169086 25918122

