# Supplementary material for: miR-381-3p Cooperated With Hes1 to Regulate the Proliferation and Differentiation of Retinal Progenitor Cells
Source: Front Cell Dev Biol. 2022 Feb 25;10:853215. doi: 10.3389/fcell.2022.853215 (PMC8914042; doi:10.3389/fcell.2022.853215)
Supplement: Supplementary file 1 [file DataSheet1.docx]

| Genes | Forward(5’-3’) | Reverse(5’-3’) | Annealing  temperature(℃) | Product size  (base pairs) |
| --- | --- | --- | --- | --- |
| Hes1 | CCAGCCAGTGTCAACACGA | AATGCCGGGAGCTATCTTTCT | 60 | 166 |
| rhodopsin | TCACCACCACCCTCTACACA | TGATCCAGGTGAAGACCACA | 60 | 216 |
| Ki-67 | CAGTACTCGGAATGCAGCAA | CAGTCTTCAGGGGCTCTGTC | 60 | 170 |
| PKC-α | CCCATTCCAGAAGGAGATGA | TTCCTGTCAGCAAGCATCAC | 60 | 212 |
| β3-tubulin | CGAGACCTACTGCATCGACA | CATTGAGCTGACCAGGGAAT | 60 | 152 |
| Brn-3a | CGCTCTCGCACAACAACATGA | TTCTTCTCGCCGCCGTTGA | 60 | 121 |
| GFAP | AGAAAACCGCATCACCATTC | TCACATCACCACGTCCTTGT | 60 | 184 |
| β-actin | AGCCATGTACGTAGCCATCC | CTCTCAGCTGTGGTGGTGAA | 60 | 152 |

**Table S1. Primer list for qPCR**


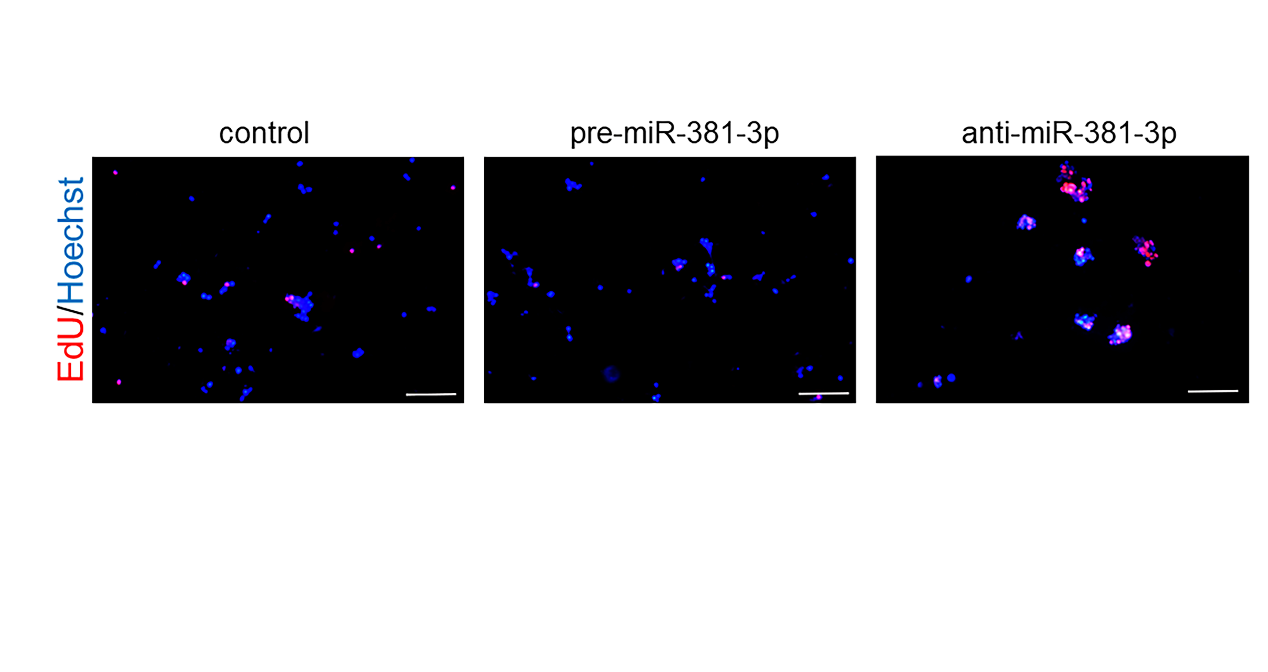


**Fig. S1. miR-381-3p inhibits RPCs proliferation.** EdU assays showed that miR-381-3p mimics attenuated proliferation in cells while miR-381-3p inhibitors significantly promoted proliferation. Scale bars: 100 µm.
